# Supplementary material for: Screening efficiency of the Mood and Feelings Questionnaire (MFQ) and Short Mood and Feelings Questionnaire (SMFQ) in Swedish help seeking outpatients
Source: PLoS One. 2020 Mar 25;15(3):e0230623. doi: 10.1371/journal.pone.0230623 (PMC7094832; doi:10.1371/journal.pone.0230623)
Supplement: S1 Table — (DOCX) [file pone.0230623.s001.docx]

## Supplementary table 1. The frequency of psychiatric disorders in the outpatient sample with Mood and Feelings Questionnaire ratings from both parent and patient (n=186).

| Psychiatric disorders | Boys  n % | | | Girls  n % | | Total  n % | |  |
| --- | --- | --- | --- | --- | --- | --- | --- | --- |
| Any depressive disorder | 28 | 27.5 | 31 | | 36.9 | 59 | 31.7 | |
| Major depression | 15 | 14.7 | 20 | | 23.8 | 35 | 18.8 | |
| Dysthymia | 2 | 2.0 | 5 | | 6.0 | 7 | 3.8 | |
| Depression NOS | 11 | 10.8 | 6 | | 7.1 | 17 | 9.1 | |
| Any anxiety^a^ | 30 | 29.4 | 34 | | 40.5 | 64 | 34.4 | |
| Any adhd^b^ | 74 | 72.5 | 40 | | 47.6 | 114*** | 61.3 | |
| Any disruptive^c^ | 37 | 36.3 | 23 | | 27.4 | 60 | 32.3 | |
| Any autism^d^ | 15 | 14.7 | 5 | | 6.0 | 20 | 10.8 | |

*** p=.001 between boys and girls.

^a^separation anxiety, social anxiety and generalized anxiety disorder,

^b^adhd combined, inattentive, hyperactive and not otherwise specified,

^c^oppositional defiant disorder, conduct disorder and disruptive disorder not otherwise specified, ^d^autistic disorder, Asperger’s disorder and Pervasive developmental disorder not otherwise specified.
