# Supplementary material for: Screening efficiency of the Mood and Feelings Questionnaire (MFQ) and Short Mood and Feelings Questionnaire (SMFQ) in Swedish help seeking outpatients
Source: PLoS One. 2020 Mar 25;15(3):e0230623. doi: 10.1371/journal.pone.0230623 (PMC7094832; doi:10.1371/journal.pone.0230623)
Supplement: S2 Table — (DOCX) [file pone.0230623.s002.docx]

## Supplementary table 2. Means, standard deviations and independent t-test for depression or non-depression for child and parent ratings on the Mood and Feelings Questionnaire (MFQ) and Short Mood and Feelings Questionnaire (SMFQ).

| **MFQ scale** | **Any depression** m (sd)  n=59 | **Non-depression** m (sd)  n=127 | **t-test** | **p-value** |
| --- | --- | --- | --- | --- |
| **Child MFQ** | 28.3 (16.0) | 17.0 (12.3) | 5.31 | < .001 |
| **Child SMFQ** | 11.9 (7.0) | 7.2 (5.5) | 5.03 | < .001 |
|  | n=69 | n=163 |  |  |
| **Parent MFQ** | 20.8 (12.8) | 11.0 (9.1) | 6.64 | < .001 |
| **Parent SMFQ** | 9.4 (5.8) | 5.1 (4.7) | 6.04 | < .001 |
