# Supplementary material for: Screening efficiency of the Mood and Feelings Questionnaire (MFQ) and Short Mood and Feelings Questionnaire (SMFQ) in Swedish help seeking outpatients
Source: PLoS One. 2020 Mar 25;15(3):e0230623. doi: 10.1371/journal.pone.0230623 (PMC7094832; doi:10.1371/journal.pone.0230623)
Supplement: S3 Table — (DOCX) [file pone.0230623.s003.docx]

### Supplementary table 3. Means, standard deviations, analysis of variance and independent t-test for Mood and Feelings Questionnaire (MFQ) and Short Mood and Feelings Questionnaire (SMFQ) with child and parent ratings separately for boys and girls and for children and adolescents.

| MFQ scale | All  m (sd)  n=186 | Pre-Boys^d^  m (sd)  n=67 | Pre-Girls^d^  m (sd)  n=34 | Teen-boys^e^  m (sd)  n=35 | Teen-girls^e^  m (sd)  n=50 | ANOVA  F | p-value |
| --- | --- | --- | --- | --- | --- | --- | --- |
| Child MFQ | 20.6 (14.5) | 14.5 (10.7)^c^ | 20.5 (13.9)^b^ | 19.8 (15.6)^b^ | 29.4 (14.6)^a^ | 11.819 | < .001 |
| Child SMFQ | 8.7 (6.4) | 6.7 (4.8)^c^ | 9.0 (5.7)^a,b^ | 7.9 (7.3)^b,c^ | 11.8 (7.0)^a^ | 6.908 | < .001 |
|  | **n=232** | **n=82** | **n=52** | **n=43** | **n=55** |  |  |
| Parent MFQ | 13.9 (11.2) | 12.3 (8.6)^b^ | 12.4 (10.6)^b^ | 17.0 (13.4)^a^ | 16.7 (13.4)^a^ | 4.868 | .003 |
| Parent SMFQ | 6.4 (5.4) | 5.8 (4.4)^b,c^ | 5.8 (5.5)^c^ | 7.9 (6.3)^a,b^ | 7.2 (6.3)^a^ | 3.161 | .025 |

^a-c^ Different superscript across groups means p< .05, same superscript means non-significant.

^d^ Pre-Boys/Girls means boys/girls 6-12 years old,

^e^ Teen-Boys/Girls means boys/girls 13-17 years old.
