# Supplementary material for: Screening efficiency of the Mood and Feelings Questionnaire (MFQ) and Short Mood and Feelings Questionnaire (SMFQ) in Swedish help seeking outpatients
Source: PLoS One. 2020 Mar 25;15(3):e0230623. doi: 10.1371/journal.pone.0230623 (PMC7094832; doi:10.1371/journal.pone.0230623)
Supplement: S4 Table — (DOCX) [file pone.0230623.s004.docx]

## Supplementary table 4. STARD checklist of reporting guidelines

|  | **Section & Topic** | **No** | **Item** | **Reported on page #** |
| --- | --- | --- | --- | --- |
|  |  |  |  |  |
|  | **TITLE OR ABSTRACT** |  |  |  |
|  |  | **1** | Identification as a study of diagnostic accuracy using at least one measure of accuracy  (such as sensitivity, specificity, predictive values, or AUC) | Page 7 (AUC, sensitivity, specificity, kappa) |
|  | **ABSTRACT** |  |  |  |
|  |  | **2** | Structured summary of study design, methods, results, and conclusions  (for specific guidance, see STARD for Abstracts) | Abstract according to STARD for abstracts |
|  | **INTRODUCTION** |  |  |  |
|  |  | **3** | Scientific and clinical background, including the intended use and clinical role of the index test | page 2-4 (intended use is in unselected clinical samples) |
|  |  | **4** | Study objectives and hypotheses | page 4 (To evaluate and compare the MFQ screening properties for age and gender and arrive at cut-offs) |
|  | **METHODS** |  |  |  |
|  | *Study design* | **5** | Whether data collection was planned before the index test and reference standard  were performed (prospective study) or after (retrospective study) | page 5-6 (prospective) |
|  | *Participants* | **6** | Eligibility criteria | page 5 (ages 6-17, not needing translator) |
|  |  | **7** | On what basis potentially eligible participants were identified  (such as symptoms, results from previous tests, inclusion in registry) | page 5 (consecutive new admissions) |
|  |  | **8** | Where and when potentially eligible participants were identified (setting, location and dates) | page 4 (four outpatient clinics) |
|  |  | **9** | Whether participants formed a consecutive, random or convenience series | page 7 (consecutive but also approving of the offered date for interview) |
|  | *Test methods* | **10a** | Index test, in sufficient detail to allow replication | page 6-7 (MFQ Swedish version) |
|  |  | **10b** | Reference standard, in sufficient detail to allow replication | page 5-6 (LEAD diagnoses based on K-SADS-PL and subsequent clinical information) |
|  |  | **11** | Rationale for choosing the reference standard (if alternatives exist) | page 7 (LEAD is considered gold standard) |
|  |  | **12a** | Definition of and rationale for test positivity cut-offs or result categories  of the index test, distinguishing pre-specified from exploratory | Page 7 (Area Under Curve for max separation and for sensitivity >.80) |
|  |  | **12b** | Definition of and rationale for test positivity cut-offs or result categories  of the reference standard, distinguishing pre-specified from exploratory | page 7 (a positive test was a LEAD diagnosis of any DSM-IV depression) |
|  |  | **13a** | Whether clinical information and reference standard results were available  to the performers/readers of the index test | page 6 (MFQ results were not accessible until after diagnoses were established) |
|  |  | **13b** | Whether clinical information and index test results were available  to the assessors of the reference standard | page 6 (Assessors were blind to MFQ but could review all other clinical information) |
|  | *Analysis* | **14** | Methods for estimating or comparing measures of diagnostic accuracy | page 7 (Receiver Operating Characteristics with sensitivity, specificity and kappa agreement) |
|  |  | **15** | How indeterminate index test or reference standard results were handled | Not applicable |
|  |  | **16** | How missing data on the index test and reference standard were handled | page 5 (MFQs were filled in completely or missing altogether) |
|  |  | **17** | Any analyses of variability in diagnostic accuracy, distinguishing pre-specified from exploratory | Page 10 (table 2 with AUC and 95% confidence intervals for boys/girls and children/adolescents) |
|  |  | **18** | Intended sample size and how it was determined | Page 4 (intended sample size was 300 in order to subgrouping for age/gender but with loss described in fig 1 we had lower power) |
|  | **RESULTS** |  |  |  |
|  | *Participants* | **19** | Flow of participants, using a diagram | page 6 (figure 1) |
|  |  | **20** | Baseline demographic and clinical characteristics of participants | page 8 and S1 table (any depression in n=59, 31.7% and in 8 of 57 (14.0%) girls 6-12 years, in 19 of 97 (19.6%) boys 6-12 years, in 21 of 53 (39.6%) boys 13-17 and in 32 of 60 (53.3%) girls 13-17 years old) |
|  |  | **21a** | Distribution of severity of disease in those with the target condition | Not reported |
|  |  | **21b** | Distribution of alternative diagnoses in those without the target condition | page 8 (other diagnoses in sample were ADHD 61.3%, anxiety disorders 34.4%, disruptive disorders 32.3% and autism spectrum disorders 10.8%) |
|  |  | **22** | Time interval and any clinical interventions between index test and reference standard | page 5-6 (K-SADS-PL as a major part of LEAD was performed at the same day MFQ was filled in and LEAD diagnosis was 1.2 sd 0.6 year later) |
|  | *Test results* | **23** | Cross tabulation of the index test results (or their distribution)  by the results of the reference standard | Not reported |
|  |  | **24** | Estimates of diagnostic accuracy and their precision (such as 95% confidence intervals) | Pages 10-12 (table 2 and 3) |
|  |  | **25** | Any adverse events from performing the index test or the reference standard | Not applicable |
|  | **DISCUSSION** |  |  |  |
|  |  | **26** | Study limitations, including sources of potential bias, statistical uncertainty, and generalisability | page 17 (data refers to help seeking patients in Sweden, with high rates of adhd and disruptive disorders. Numbers are low for subgrouping age/gender) |
|  |  | **27** | Implications for practice, including the intended use and clinical role of the index test | page 16 (SMFQ is suggested for clinical screening in adolescents and with gender specific cut-offs while use in children is not recommended) |
|  | **OTHER INFORMATION** |  |  |  |
|  |  | **28** | Registration number and name of registry | Not applicable |
|  |  | **29** | Where the full study protocol can be accessed | Not reported |
|  |  | **30** | Sources of funding and other support; role of funders | on page 17 |
|  |  |  |  |  |
