# Supplementary material for: Screening efficiency of the Mood and Feelings Questionnaire (MFQ) and Short Mood and Feelings Questionnaire (SMFQ) in Swedish help seeking outpatients
Source: PLoS One. 2020 Mar 25;15(3):e0230623. doi: 10.1371/journal.pone.0230623 (PMC7094832; doi:10.1371/journal.pone.0230623)
Supplement: Attachment — Submitted filename: Response to Reviewers.docx [file pone.0230623.s006.docx]

Response to Reviewers

## Reviewer #1

1. **Background on depression in children.** Good point which we had omitted. We have now added that prevalence is considerably lower and not increasing in children while we also added that the degree of clinical recognition in children is not known.
2. **Role of the K-SADS-PL in the introduction section.** We have specified the different criterion test that arrived at opposing cut offs (same for boys or girls versus a lot higher cut off for girls). The criterion was a clinician diagnosis utilising K-SADS-PL in one study while the criterion was just a cut-off on a self report in the other study. Thus, the role of K-SADS as a more reliable diagnostic test than a self report form is explained.
3. **Aims of study as clear separate segments.** The aims are now separated by a line break and are specified as just two and more clearly phrased as ”to …”
4. **To describe the reason for attrition more clearly.** A more detailed description of the inclusion process are included as well as a specification of the missing 35 parental MFQ was due to administrative problems.
5. **Criterion scale in the study.** Very important point and possible ”reference test” was not a clear description of the role of LEAD in this major design issue. We have now clearly stated in several segments (aims, statistics, results…heading of table 2 and discussion) that a LEAD diagnosis was the criterion in the study. However, it is not possible to describe the validity of the LEAD while the interrater reliability of the LEAD is now described in the methods section.

## Reviewer #2

1. **Validity of the MFQ and SMFQ in the methods section.** As the validity of the scale is the main focus of the paper, we chose to describe and discuss this topic in the introduction. This discussion is the base for the summing up of the background and aims of the study. Therefor, we would still prefer to describe previous research on validity of MFQ in the introduction.
2. **A final conclusion (same as in abstract) is added.** Thanks for this suggestion!
3. **References before 2000.** We would like to keep these references as the original studies on MFQ were published in the late 90´s. However, we are refering to just about every relevant study MFQ and SMFQ including all with more recent dates.
